# Outgrowth, proliferation, viability, angiogenesis and phenotype of primary human endothelial cells in different purchasable endothelial culture media: feed wisely

**DOI:** 10.1007/s00418-019-01815-2

**Published:** 2019-09-21

**Authors:** Barbara Leopold, Jasmin Strutz, Elisa Weiß, Juergen Gindlhuber, Ruth Birner-Gruenberger, Hubert Hackl, Hannah M. Appel, Silvija Cvitic, Ursula Hiden

**Affiliations:** 1 Department of Obstetrics and Gynaecology, Medical University of Graz, Graz, Austria; 2 Institute of Pathology, Medical University of Graz, Graz, Austria; 3 Omics Center Graz, BioTechMed-Graz, Graz, Austria; 4 Institute of Chemical Technologies and Analytics, Vienna University of Technology, Vienna, Austria; 5 Division of Bioinformatics, Biocenter, Medical University of Innsbruck, Innsbruck, Austria

**Keywords:** Primary endothelial cells, Culture media, Outgrowth, Proliferation, Viability, Angiogenesis

## Abstract

Function and dysfunction of endothelial cells are regulated by a multitude of factors. Endothelial cell research often requires in vitro cell culture experiments. Hence, various culture media specifically designed to promote endothelial cell growth are available. These strikingly differ in their composition: complex media contain endothelial cell growth supplement (ECGS), an extract produced of bovine brain with undefined amounts of biologically active compounds, whilst defined media contain selected growth factors in defined concentrations. We here compared the effect of seven purchasable endothelial cell culture media on colony outgrowth, proliferation, viability, in vitro angiogenesis and phenotype of mature primary human endothelial cells using feto-placental endothelial cells isolated from chorionic arteries (fpEC). The effect of media on colony outgrowth was additionally tested on umbilical cord blood-derived endothelial progenitor cells (ECFCs). Outgrowth, purity, proliferation and viability differed between media. Outgrowth of fpEC and ECFCs was best in a defined medium containing EGF, FGF2 and VEGF. By contrast, established fpEC isolations proliferated best in complex media containing ECGS, heparin and ascorbic acid. Also viability of cells was higher in complex media. In vitro angiogenesis was most intense in a defined medium containing the highest number of individual growth factors. FACS analysis of surface markers for endothelial cell subtypes revealed that endothelial phenotype of fpEC was unaffected by media composition. Our data demonstrate the fundamental effect of endothelial cell culture media on primary cell isolation success and behaviour. Whether the composition of supplements is suitable also for individual experiments needs to be tested specifically.

**Electronic supplementary material:**

The online version of this article (10.1007/s00418-019-01815-2) contains supplementary material, which is available to authorized users.

## Introduction

Endothelial cells are highly versatile and their various functions are regulated by a multitude of factors either present in the circulation, or available locally in the microenvironment. Endothelial functions include vascular tone regulation, establishment of a barrier, leukocyte transmigration, blood coagulation and angiogenesis. Endothelial function and dysfunction are involved in inflammation, in the development of cardiovascular disease and long-term complications of diabetes, and in tumour growth which is driven by exaggerating angiogenesis. Thus, endothelial cell culture is an important tool in various research areas. This is highlighted by the number of scientific publications using endothelial cells in vitro: the search term ‘endothelial cell culture’ reveals more than 1000 publications released in the past 12 months in PubMed.

Various endothelial cell phenotypes exist which differ depending on their vascular origin (Aitsebaomo et al. 2008). For instance, arteries and veins differ regarding Ephrin, Notch and VEGF signalling, but furthermore, organ-specific differences in endothelial functions and phenotypes exist (Aitsebaomo et al. 2008). Moreover, endothelial phenotype depends on the differentiation state: endothelial progenitor cells are characterized by expression of CD34, CD45 (protein tyrosine phosphatase, receptor type C, PTPRC), CD90 (Thy-1 cell surface antigen, THY1) and CD133 (prominin 1, PROM1), whilst CD144 (vascular endothelial cadherin, CDH5) and CD146 (melanoma cell adhesion molecule, MCAM) are typically expressed on mature, vascular endothelial cells (Rakocevic et al. 2017). CD31 (platelet and endothelial cell adhesion molecule, PECAM1) and CD309 (vascular endothelial growth factor receptor 2, VEGFR2) are expressed on both progenitor and mature endothelial cells [reviewed by Timmermans et al. (2009) and Rakocevic et al. (2017)].

Basically, primary endothelial cells can be either isolated from dissected blood vessels and vessels located within tissue specimen, or from the pool of endothelial progenitor cells circulating in the bloodstream. For the isolation of mature endothelial cells from established blood vessels, most protocols rely on enzymatic detachment and release of single endothelial cells. Large vessels, such as the umbilical vein or chorionic arteries, can be directly perfused by the enzyme solution to obtain single endothelial cells (Ganguly et al. 2012; Lang et al. 2008). Isolation of endothelial cells from whole tissue specimen requires immunopurification of endothelial cells after the enzymatic treatment. Immunopurification may be performed using magnetic beads (Zabini et al. 2012) or FACS sorting (Naschberger et al. 2018). A further source of endothelial cells is the pool of circulating progenitors in the bloodstream. Their isolation does not require enzymatic treatment and endothelial cells are separated from other mononucleated cells by their ability to attach to the culture dish and to form colonies. Thus, irrespective of the isolation method, the attachment of the cells to the culture dish and the initiation of proliferation are crucial steps in primary endothelial cell culture, and the quality, purity and quantity of colony outgrowth are central for subsequent in vitro studies.

After successful isolation, further in vitro expansion of the cells for subsequent experiments depends on their proliferation and viability. In vivo, proliferation of endothelial cells for vascular growth and angiogenesis is restricted to embryonic and placental development, and to a few processes later in life, including wound healing and menstrual cycle [reviewed by Carmeliet (2005)]. Hence, vascular endothelial cells are mostly quiescent and do not undergo cell cycle progression. However, during in vitro cell culture, endothelial cells are required to proliferate and to expand for experimental use. Thus, endothelial cell culture uses specialized culture media supplemented with a multitude of growth factors to stimulate endothelial cell proliferation, but, at the same time, without affecting endothelial phenotype or function. In fact, the optimisation of culture conditions for proliferation of endothelial cells is a recurrent concern in research (Engelmann and Friedl 1989; Friedl et al. 1989; Peh et al. 2013; Proulx et al. 2007; Terramani et al. 2000).

Various endothelial culture media are available, which fundamentally differ in the composition of growth supplements. Whilst some media are supplemented with defined concentrations of recombinant growth factors, such as EGF (epidermal growth factor), FGF2 (fibroblast growth factor 2), VEGF (vascular endothelial growth factor) and IGF1 (insulin-like growth factor 1), other media contain endothelial cell growth supplements (ECGS) that are produced of bovine brain (BBE), pituitary (BPE) or hypothalamus (BHE) and are rich in growth-promoting molecules of undefined composition and quantity. Besides growth factors, endothelial cell media can be supplemented with hydrocortisone or may contain l-glutamine, heparin, ascorbic acid and cyclic AMP. Since endothelial cell growth, proliferation, viability and differentiation are regulated by endocrine factors (Minehata et al. 2002), they may also be modulated by culture media and supplements.

We here aimed to evaluate the properties of different commercially available endothelial culture media on outgrowth, proliferation and function of primary human endothelial cells. Therefore, we employed our model of primary human arterial feto-placental endothelial cells (fpEC) (Lang et al. 2008; Loegl et al. 2016, 2017) and investigated fpEC colony outgrowth after isolation, proliferation, viability, in vitro angiogenesis and endothelial phenotype in seven different purchased defined and complex culture media. Since we observed a very distinct effect of colony outgrowth and isolation success between the various media, we further tested their effect on outgrowth of endothelial colony-forming cells (ECFCs) isolated from umbilical cord blood.

## Materials and methods

### Endothelial cell culture media

The commercially available culture media specifically developed for endothelial cell culture were purchased from ATCC (Manassas, VA, USA), LifeLine Celltech (Frederick, MD, USA), Lonza (Walkersville, MD, USA), PromoCell (Heidelberg, Germany), Cell Applications (San Diego, CA, USA), PAN-Biotech (Aidenbach, Germany) and Gibco (Paisley, UK) (Table 1). The undefined, complex media contained endothelial cell growth supplement (ECGS), a poorly defined supplement containing various growth factors, such as bovine brain extract (BBE), bovine pituitary extract (BPE) and bovine hypothalamic extract (BHE). The defined media contained specified concentrations of growth factors, such as epithelial growth factor (EGF), fibroblast growth factor 2 (FGF2), vascular endothelial growth factor (VEGF) or insulin-like growth factor 1 (IGF1). Composition of culture media is summarized in Table 2.

**Table 1:** Company, specific media and supplements tested in this study

Company	Basal medium	Supplement	Medium identifier
ATCC	Vascular Cell Basal M.	Microvascular EC Growth Kit-BBE	ATCC
LifeLine Celltech	VascuLife Basal M.	VascuLife EnGS-Mv LifeFactors Kit	VascuLife
Lonza	EC Basal M.	EGM-MV Bullet Kit	EGM-MV
PromoCell	EC M. MV	EC Growth M. MV Suppl. Pack	PromoCell
Cell Applications	Human Microvasc. EC Basal M.	Human Microvascular EC Growth Suppl.	Cell Appl.
PAN-Biotech	Endopan MV Microvasc. EC Basal M.	Endopan MV Kit	Endopan
Gibco	MCDB 131 M.	Microvasc. Growth Suppl.	MCDB 131

The medium identifier is the term used for the respective medium throughout this study

*M.* Medium, *EC* endothelial cell, *Suppl*. Supplement

**Table 2:** Composition of endothelial cell media tested in this study

Compound	Complex media				Defined media		
	ATCC	VascuLife	EGM-MV	PromoCell	Cell Appl.	Endopan	MCDB 131
ECGS	0.2% (BBE)	0.2% (n. s.)	0.4% (BBE)	0.4% (BHE)			
EGF	5 ng/ml	5 ng/ml	+	10 ng/ml	+	+	1 ng/ml
FGF2					+	+	3 ng/ml
VEGF					+	+	
IGF1						+	
Hydrocortisone	1 µg/ml	1 µg/ml	+	1 µg/ml		+	1 µg/ml
l-Glutamine	10 mM	10 mM					
Heparin	0.75 U/ml	0.75 U/ml		90 µg/ml			10 µg/ml
Ascorbic acid	50 µg/ml	50 µg/ml				+	
FBS	5%	5%	5%	5%	5%	6%	4.9%
Dibutyryl cyclic AMP							0.08 mM
Antibiotics	Optional	Optional	+	Optional	Optional	+	Optional

*n.s.* not stated, *ECGS* endothelial cell growth supplement such as bovine brain extract (BBE), bovine pituitary extract (BPE) and bovine hypothalamic extract (BHE). *FBS* fetal bovine serum. *+* indicates the presence of the respective supplement, but without concentration stated by the manufacturer

### Isolation and culture of primary arterial feto-placental endothelial cells (fpEC)

Isolation of fpEC from placental chorionic vessels was approved by the ethics committee of the Medical University of Graz (29-319 ex 16/17). Written informed consent was obtained. Directly after delivery, primary fpEC were isolated as described by Lang et al. (2008). Placentas were collected after caesarean sections or spontaneous deliveries. Pieces (3 cm) of arterial chorionic plate vessels were dissected and washed with 1 × Hanks Balanced Salt Solution (HBSS, Thermo Fisher Scientific, MA, USA). Then, arteries were cannulated using a 20 gauge needle (Smiths Medical) and perfused with 20 ml of prewarmed (37 °C) 0.5% Collagenase/Dispase solution (Roche, Mannheim, Germany) for 8 min (flow rate 2.5 ml/min). The released cells were collected in a tube containing 10 ml fetal calf serum (FCS, HyClone, GE Healthcare) and centrifuged for 7 min at 900 rpm at room temperature (RT). Cells were resuspended in 1 ml of the respective media. Only during the first culture outgrowth, FCS was replaced by 10% human pooled and heat inactivated serum (Atlanta Biologicals, Flowery Branch, GA, USA). This was not possible for the media from Gibco and Cell applications which provided all supplements premixed. The cell suspension isolated from one individual vessel was seeded per well in a 12-well culture plate (Corning, NY, USA) pre-coated with 1% porcine skin gelatine (Sigma-Aldrich, St. Louis, MO, USA). Wells were observed daily and medium was changed after 2 days and then twice a week. After reaching confluency, fpEC were transferred into 12.5 cm^2^ culture flasks and human serum was replaced by FCS according to the original media composition. Cells were then further expanded and subjected to immunocytochemical characterization for identity and purity as described below. fpEC isolations were cultured at 12% O_2_, 5% CO_2_ and 37 °C in a 90% humidified incubator.

### Isolation and culture of endothelial colony-forming cells (ECFCs) from umbilical cord blood

Isolation of ECFCs from umbilical cord blood was approved by the ethics committee of the Medical University of Graz (29-319 ex 16/17). Written informed consent was obtained. ECFCs were isolated as described (Tasev et al. 2018): immediately after delivery, 10–15 ml of umbilical cord blood was collected in lithium heparin pre-coated tubes (Greiner Bio One, Kremsmünster, Austria) by puncture of the umbilical vein. Mononuclear cells (MNCs) were separated from other blood components by density gradient centrifugation. Therefore, umbilical cord blood was diluted with HBSS to a final volume of 30 ml and layered on top of 15 ml Lymphoprep™ Density Gradient Medium (Axis Shild, Alere Technologies AS, Oslo, Norway). After centrifugation at 2000 rpm at RT for 20 min without brake, the layer containing MNCs was transferred into a fresh tube, washed with HBSS and centrifuged at 1300 rpm for 5 min. The cell pellet was resuspended in HBSS and cell number was determined using Casy-TT cell counter. MNCs (2 × 10^7^ cells) were seeded in 2 ml culture medium per well in a 6-well culture plate pre-coated with 5 µg/cm^2^ rat tail collagen type 1 (Corning, Bedford MA) in 0.02 M acetic acid (Merck Millipore, MA) and cultured at 21% O_2_, 5% CO_2_ and 37 °C in a 90% humidified incubator. After 24 h, the medium was changed to remove non-adherent cells and then renewed twice a week. ECFC colonies were identified as monolayers of cells with cobblestone morphology and counted every day. After 13 days, cells were expanded and subjected to immunocytochemical characterisation as described below.

### Immunocytochemical characterisation

The presence of the endothelial cell markers CD31 and von Willebrand factor (VWF) and the complete absence of cells expressing the fibroblast marker CD90 and the smooth muscle cell marker smooth muscle actin (SMA) was used as confirmation of endothelial cell identity. These proteins were detected using Ultra Vision HRP (horseradish peroxidase) Polymer Kit (Thermo Scientific, Rockford, IL).

Cells (100,000 per 1.7 cm^2^ chamber) were grown in the respective media on glass chamber slides for 24 h. Then, cells were washed with HBSS dried over night at RT and fixed with ice-cold acetone (Merck, Darmstadt, Germany) for 3 min. All subsequent steps were performed at RT. Slides were rehydrated in TBE pH 7.5 with 0.1% Tween (Sigma, St. Lois, MO) for 3 min, which was also used as a washing puffer. Primary antibodies (Table 3) and negative controls of the same isotype and in the same dilution (Dako, Glostrup, Denmark) were applied and incubated for 30 min. Cells were washed and primary antibody enhancer was applied for 10 min, followed by further washing. Subsequently, HRP Polymer (a polymer conjugate containing multiple HRP molecules) was applied and incubated for 15 min in the dark. After washing, cells were incubated with a peroxidase-compatible chromogen (Thermo Scientific) for 5 min and washed again with distilled water. Cells were counterstained with hematoxylin and mounted with Aquatex (Merck).

**Table 3:** Antibodies and isotype controls used for immunocytochemistry

Antibody	Clone	Manufacturer/order no.	Isotype	Dilution
CD31	EN4	Monosan/MON6002-1	Mouse IgG1	1:300
vWF	Polyclonal	Dako/A0082	Polyclonal rabbit	1:3000
SMA	1A4	Dako/M0851	Mouse IgG2a	1:200
CD90	AS02	Dianova/DIA100	Mouse IgG1	1:100

### BrdU cell proliferation ELISA

DNA synthesis as a measure of proliferation was performed using a colorimetric cell proliferation ELISA (Roche) based on the incorporation of 5′-bromo-2′-deoxyuridine (BrdU) during DNA replication. The ELISA was accomplished according to the manufacturer’s instructions with cell-specific adaptations. Isolated fpEC cells were cultured in the respective media for 7 days. Then, fpEC were seeded in a 96-well plate in the densities of 30,000 and 60,000 cells/cm^2^, again in the respective media (100 µl). After 21 h, 10 µl of BrdU solution (100 µM) was added to each well. After 3 h, medium was removed and cells were fixed with 200 µl acidic ready-to-use FixDenat solution for 30 min at RT. Subsequently, 100 µl of the solution containing the monoclonal anti-BrdU antibody conjugated with peroxidase were added and incubated for 90 min at RT in the dark. Each well was washed three times with 250 µl washing solution. Then, 100 µl of substrate solution was added and incubated in the dark for 15 min and absorbance was measured in a SPECTROstar Nano absorbance reader (BMG Labtech, Ortenburg, Germany) at 370 nm and at a reference wavelength of 492 nm. Experiments were performed with six independent fpEC isolations, in quadruplicates.

### Proliferation and viability assay using CASY cell counter

Isolated fpEC cells were cultured in the respective media for 7 days. Then, fpEC cells (20,000/cm^2^) were seeded in gelatine-coated 6-well plates in the respective endothelial cell media and cultured for 24 and 48 h. Cell number and viability were determined with a Casy-TT Analyser System (OLS, Omni Life Science, Bremen, Germany) according to the manufacturer’s instructions (Wang et al. 2014). In brief, media were removed, cells were washed with HBSS and detached with 500 µl TrypLE™ (recombinant cell-dissociation enzymes, Thermo Fisher Scientific) at 37 °C for 5 min. Then, 500 µl of EGM-MV medium was added and the cell suspensions were transferred into 1.5 ml tubes (Eppendorf, Hamburg, Germany). Cell suspensions were diluted in Casy electrolyte solution (Casyton, 1:100) and cells were analysed using the Casy-TT cell counter in a dual measurement mode. Calibration with dead and vital fpEC cells revealed positions for the normalisation cursors at 8.00 and 48.88 µm, and for the evaluation cursors at 13.00 and 50.00 µm, allowing discrimination between cell debris, dead and living cells (Wang et al. 2014). Experiments were performed using four independent fpEC isolations, in triplicates.

### Fibrin angiogenesis assay

Fibrin assay was performed as described (Koolwijk et al. 1996). Fibrin (CoaChrom Diagnostica, Maria Enzersdorf, Austria) was dissolved in M199 medium (Lonza) in a concentration of 4 mg/ml. Then, thrombin IIa (CoaChrom Diagnostica) was added (0.03 U/ml) and the solution was immediately pipetted (100 µl/well) in a 96-well plate. For polymerisation, the plate was incubated for 1 h at RT followed by 1 h incubation at 37 °C. For thrombin inactivation, the respective media in the original composition as well as additionally supplemented with 10% newborn calf serum (NBCS, Thermo Fischer Scientific) and 5% human serum (Atlanta Biologicals) were added to each well and the plate was incubated for 2 h at 37 °C. Routinely, we use M199 medium supplemented with 10% NBCS and 10% human serum for the fibrin angiogenesis assay, therefore it served here as a control medium. Then, endothelial cells (35,000/well in 100 µl of the respective media) were seeded onto the fibrin matrix. After overnight incubation, medium was removed and replaced by 100 µl of the respective media supplemented with 10 ng/ml TNFα (ReliaTech, Wolfenbüttel, Germany) or a combination of TNFα (10 ng/ml), FGF2 (10 ng/ml; ReliaTech) and VEGF (25 ng/ml; ReliaTech) to stimulate angiogenesis. After 24 h, the fibrin assay was terminated by removing the media and addition of 100 µl warm 4% formaldehyde (DonauChemie, Wien, Austria) to each well for 2 h. Then, the formaldehyde was aspirated, wells were filled with 200 µl PBS (Medicargo, Uppsala, Sweden) and the plate was sealed with parafilm and stored at 4 °C until imaging.

### Image acquisition and tube length analysis

Z-stacks of each well were acquired with a step size of 10 µm using a Nikon TiE-2 microscope equipped with an Andor Zyla 4.2 sCMOS camera and Nikon Plan Apoλ 4x objective. The acquisition was automated with the JOBS module of NIS Elements software (vers. 5.20.01) which also included a brightness adjustment, target intensity of 75% and over illumination tolerance of 0.1%, to counteract the varying opacity of different media. Prior to analysis, stacks were reduced with the GA3 module to best focus plane and a flat field correction was performed. The analysis was executed in FIJI (vers 1.51 h). For each image, a ROI (region of interest) was drawn manually to exclude out-of-focus areas, fixation artefacts or areas showing onset of fibrin lysis. Within the ROI, automated histogram stretching and thresholding were performed. The binary mask was skeletonized and particles were analysed excluding objects of less than 150 µm to minimize unspecific background. Supplementary Fig. 1 shows the structures identified by the software in the analysis. All results were normalized to total ROI size. Data were exported to Microsoft EXCEL and used for statistical analysis.

### Flowcytometric analysis

fpEC were cultured in the different media for 7 days, detached using TrypLE™, washed with HBSS and Fc receptors were blocked for 10 min with 3% FCS in HBSS. After centrifugation, cells were resuspended in staining buffer (PBS (Thermo Fisher Scientific, MA, USA) containing 0.1% BSA (Sigma-Aldrich) and 20 mM EDTA (Thermo Fisher Scientific)) in a density of 10^6^ cells/ml. For surface staining, 100 µl of cell suspension was concomitantly stained either with the fluorochrome-conjugated primary antibody combination a, with the fluorochrome-conjugated primary antibody combination b, or with a respective combination of isotype controls corresponding to each fluorochrome (Table 4) and incubated in the dark at 4 °C for 20 min. Then, cells were washed with 1 ml staining buffer, centrifuged at 300*g* for 5 min and resuspended in 250 µl staining buffer. Measurement was performed on a CytoFLEX flow cytometer (Beckman Coulter) by the use of the associated CytExpert software (Beckman Coulter). Experiments were performed with three different fpEC isolations.

**Table 4:** Antibodies and isotype controls used for FACS analysis

Marker	Label	Manufacturer/order no.	Isotype	Volume (µl)
Antibody combination a				
CD14	FITC	Miltenyi Biotec/130-080-701	Mouse IgG2a	2
CD45	PE	BD Pharmingen/555483	Mouse IgG1k	5
CD133	APC	Miltenyi Biotec/130-098-829	Mouse IgG1k	10
CD34	PE-Cy7	Beckman Coulter/A21691	Mouse IgG1	4
Antibody combination b				
CD31	FITC	BD Pharmingen/560984	Mouse IgG1k	5
CD146	PE	BD Pharmingen/561013	Mouse IgG1k	7
CD90	APC	BD Pharmingen/561971	Mouse IgG1k	0.1
CD309	PE-Cy7	Biolegend/359912	Mouse IgG1k	5
CD144	Horizon V421	BD Pharmingen/565670	Mouse IgG1k	3
Isotype controls				
	FITC	BD Pharmingen/555748	Mouse IgG1k	
	PE	BD Pharmingen/554680	Mouse IgG1k	
	APC	BD Pharmingen/555751	Mouse IgG1k	
	PE-Cy7	Beckman Coulter/737662	Mouse IgG1	
	Horizon V421	Biolegend/562438	Mouse IgG1k	

### Statistics

Statistics was performed using GraphPad Prism version 5.0 for Windows (GraphPad Software, San Diego California USA). Contingency (successful vs unsuccessful) of fpEC isolations in different media was determined by calculating Chi square. The number of days until outgrowth was analysed using one-way ANOVA, the number of colonies was analysed using one-way (fpEC) or two-way ANOVA (ECFCs). For analysis of ECFC colony number, the means of 4–6 replicates per cell isolation and medium were used. Analysis of proliferation and viability in different media employed two-way ANOVA (repeated measures), using the mean of the quadruplicates or triplicates, respectively. Number of tubes was analysed by two-way ANOVA using the means of the triplicates per cell isolation. One and two-way ANOVA were followed by Bonferroni post hoc test.

## Results

### Effect of culture media on fpEC colony outgrowth

**Fig. 1:**
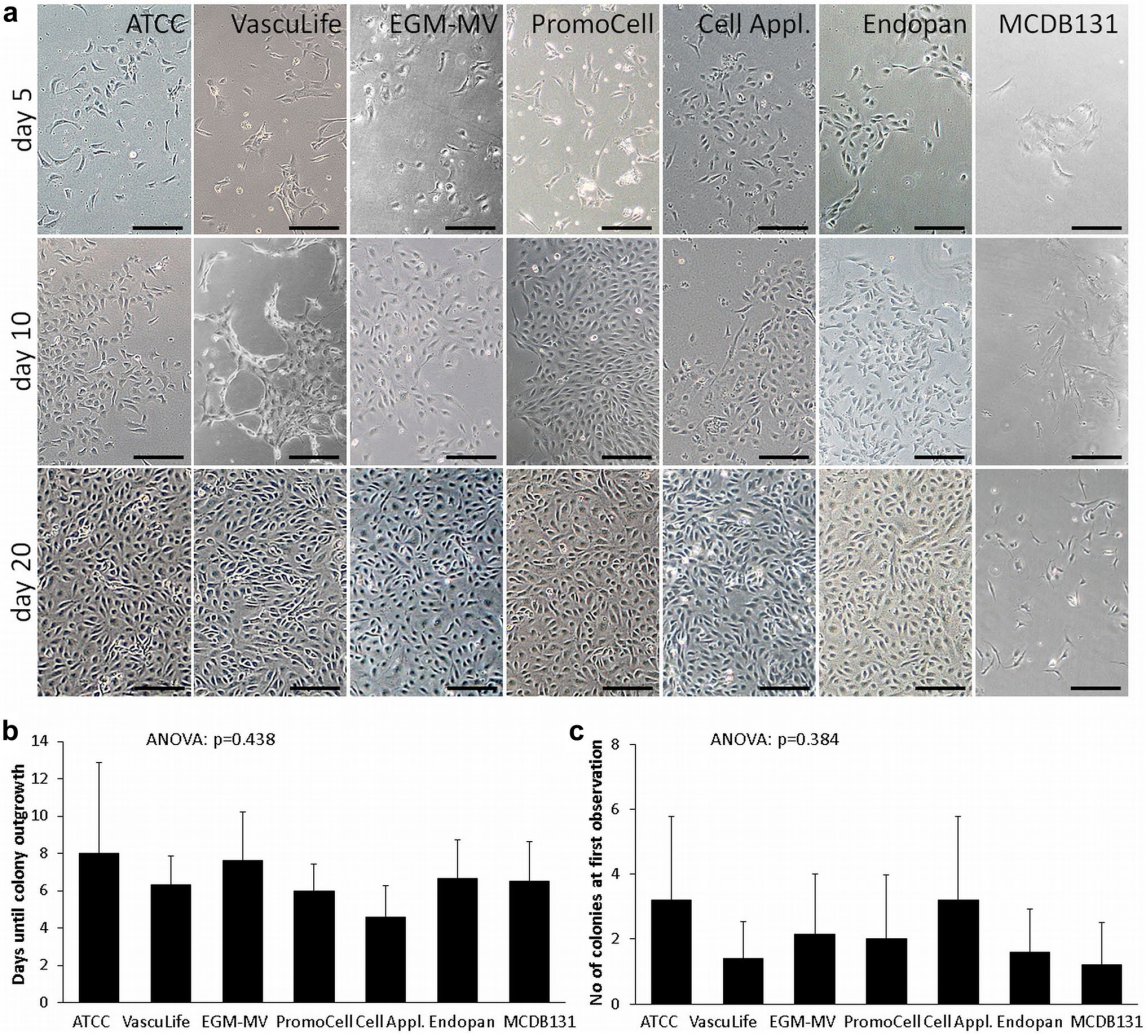
Effect of different endothelial culture media on colony outgrowth of fpEC. **a** Colony outgrowth after isolation procedure. Images were taken on day 5 (upper images), 10 (middle images) and 20 (lower images) after isolation using an Olympus CKX41 microscope (× 40 magnification). Scale bar: 200 µm. **b** Number of days until observation of first colonies after isolation. **c** Number of colonies at the first day of their observation. Data are given as mean ± SD of five (ATCC; VascuLife; Endopan; MCDB 131) or ten (EGM-MV; PromoCell; Cell Applications) fpEC isolations

Primary fpEC were isolated using seven commercially available endothelial cell culture media (Tables 1, 2). About 5 days after isolation, first scattered fpECs could be observed attached to the culture plate. These formed fpEC colonies after 6–8 days (Fig. 1a, b). Whilst in most media the morphology of colonies was similar, fpEC colonies possessed a distinct, more network-like growing pattern at the colony borders in VascuLife medium (Fig. 1a). After reaching confluency, this difference disappeared.

The period of time between isolation and first colony outgrowth was shortest in medium from Cell Applications, but without reaching significance (Fig. 1b). Also the number of colonies observed on the first day of colony outgrowth was not significantly different between media, but highest in media from ATCC and Cell Applications (Fig. 1c).

**Fig. 2:**
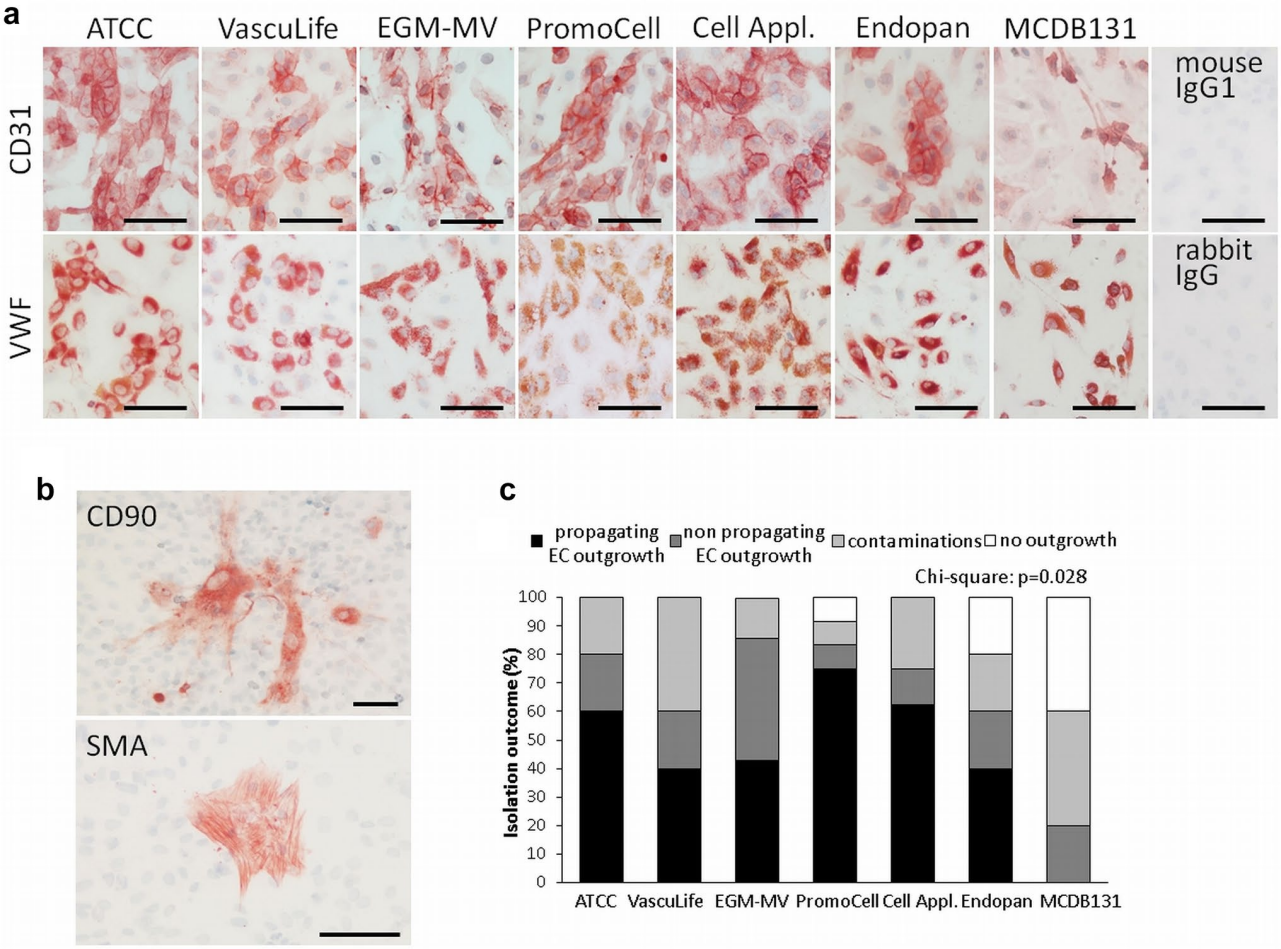
Effect of different endothelial culture media on quality of fpEC isolations. Immunocytochemistry for quality control of fpEC isolated in different culture media. Cells were seeded in chamber slides after expansion of first colonies. **a** fpEC were positive for the endothelial cell markers CD31 and VWF, with highest staining when cells attached very close to each other. **b** Contaminations with fibroblasts and smooth muscle cells were identified by positive staining for CD90 and SMA, respectively. For negative controls unspecific antibodies of the same isotype were used. Scale bar: 100 µm. **c** Overview of isolation success, i.e., % isolations without any colony outgrowth, % of outgrowth with contaminations, % of endothelial cell isolations that discontinued to proliferate and % of successful fpEC isolations, as revealed after expansion of the colonies and quality control by immunocytochemical characterisation. Contingency test (Chi square) of successful vs unsuccessful isolations revealed an overall effect of the media with a significance of 0.028. Data are given as % of total numbers of five (ATCC; VascuLife; Endopan; MCDB 131) or ten (EGM-MV; PromoCell; Cell Applications) fpEC isolations

The obtained colonies were further expanded, endothelial identity was verified and purity determined by positive immunocytochemistry for the endothelial cell markers von Willebrand factor (VWF) and CD31 (Fig. 2a) and negative staining for smooth muscle actin (SMA) and the fibroblast marker CD90, which demonstrate contamination outgrowth (Fig. 2b). Some endothelial cell isolations did not propagate further, ceased proliferation, and thus, did not give rise to expandable vital endothelial cell isolations. The isolation success, i.e., pure and expandable fpEC isolations vs isolations without any outgrowth, with contamination outgrowth, or with fpEC outgrowth that did not propagate further, differed between media (Fig. 2c): media from ATCC, PromoCell and Cell Applications gave the best results, i.e., the highest number of propagating EC isolations. No pure and propagating endothelial cells could be isolated using MCDB 131 medium.

### Effect of culture media on fpEC proliferation and viability

**Fig. 3:**
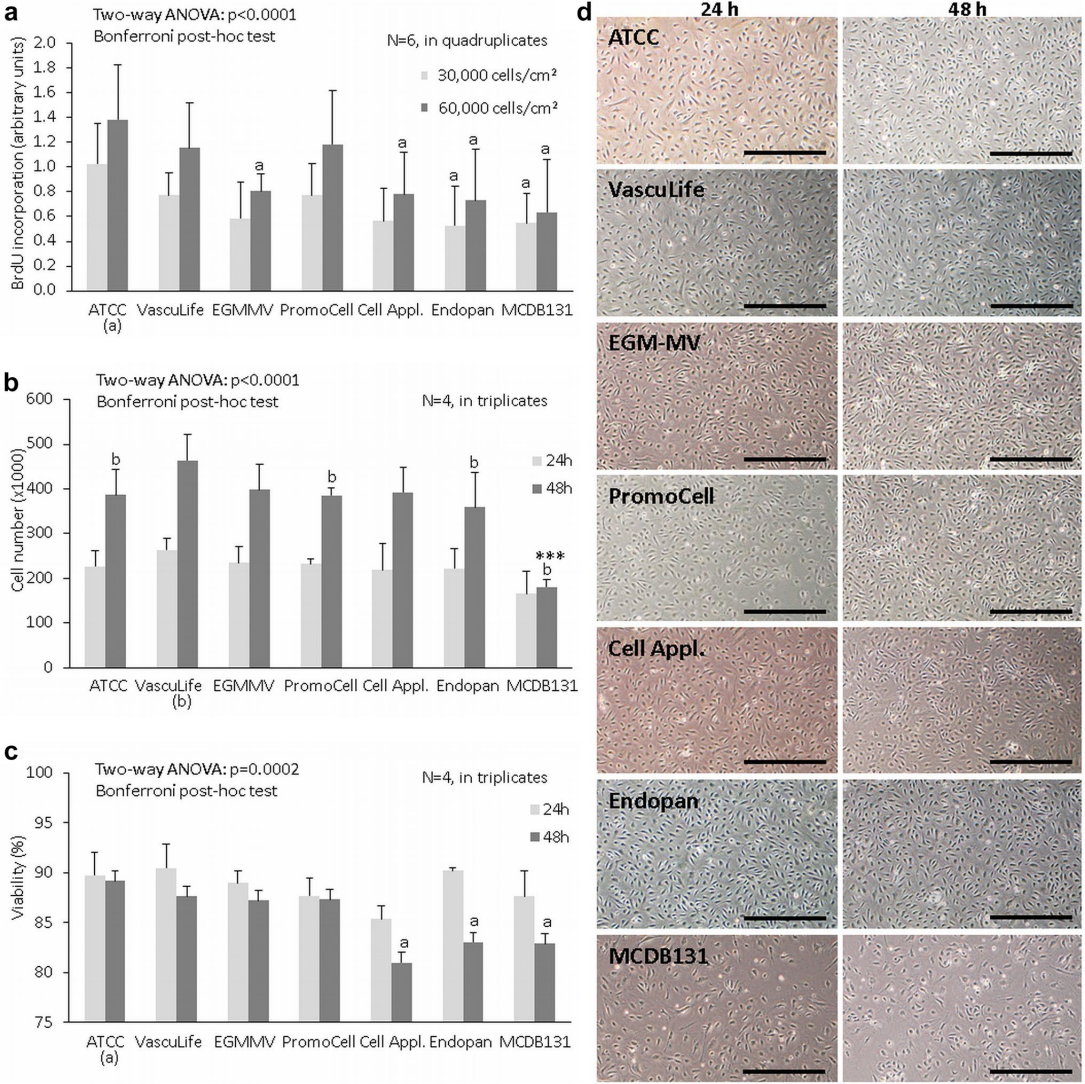
Effect of different endothelial culture media on proliferation and viability of fpEC. DNA synthesis was determined 20 h after seeding of cells in two different cell densities (30,000 vs 60,000 cells/cm^2^) using a BrdU ELISA (**a**). Cell number (**b**) and viability (**c**) were analysed 24 and 48 h after seeding using a Casy-TT instrument. Prior to counting, cells were photographed using an Olympus CKX41 microscope (× 40 magnification) (**d**). BrdU ELISA was performed with six independent cell isolations in quadruplicates. Casy analysis was performed with four independent cell isolations in triplicates. Data are given as mean ± SD. *a* significant vs respective condition in ATCC, *b* significant vs respective condition in VascuLife, ***significant vs all other media. Scale bar: 500 µm

Using established and pure fpEC isolations, we tested the effect of different culture media on proliferation and viability employing two different methods, i.e., BrdU ELISA and automated cell counting. BrdU ELISA measured DNA synthesis and thus, quantified only cells which underwent S phase in between the addition of BrdU and the measurement 3 h later. Casy cell analysis counted all cells, regardless of cell cycle phase, after 24 and 48 h. Both approaches revealed that cell proliferation and viability were higher in complex than in the defined media. The media which promoted cell proliferation best were the ones from ATCC (BrdU incorporation, Fig. 3a) and VascuLife (cell counting, Fig. 3b).

Besides cell counting, the Casy-TT instrument enables measurement of cell viability based on cell size. Medium effects on viability became evident only 48 h after cell seeding. At that time, fpEC grown in complex media revealed higher viability than fpEC grown in defined media, but this difference reached significance only for the ATCC medium (Fig. 3c).

Visual inspection of cells after 24 and 48 h revealed typical cobblestone morphology of fpEC in all media and illustrated the low cell number when cells were cultured in MCDB 131 (Fig. 3d).

### Effect of culture media on fpEC tube formation

**Fig. 4:**
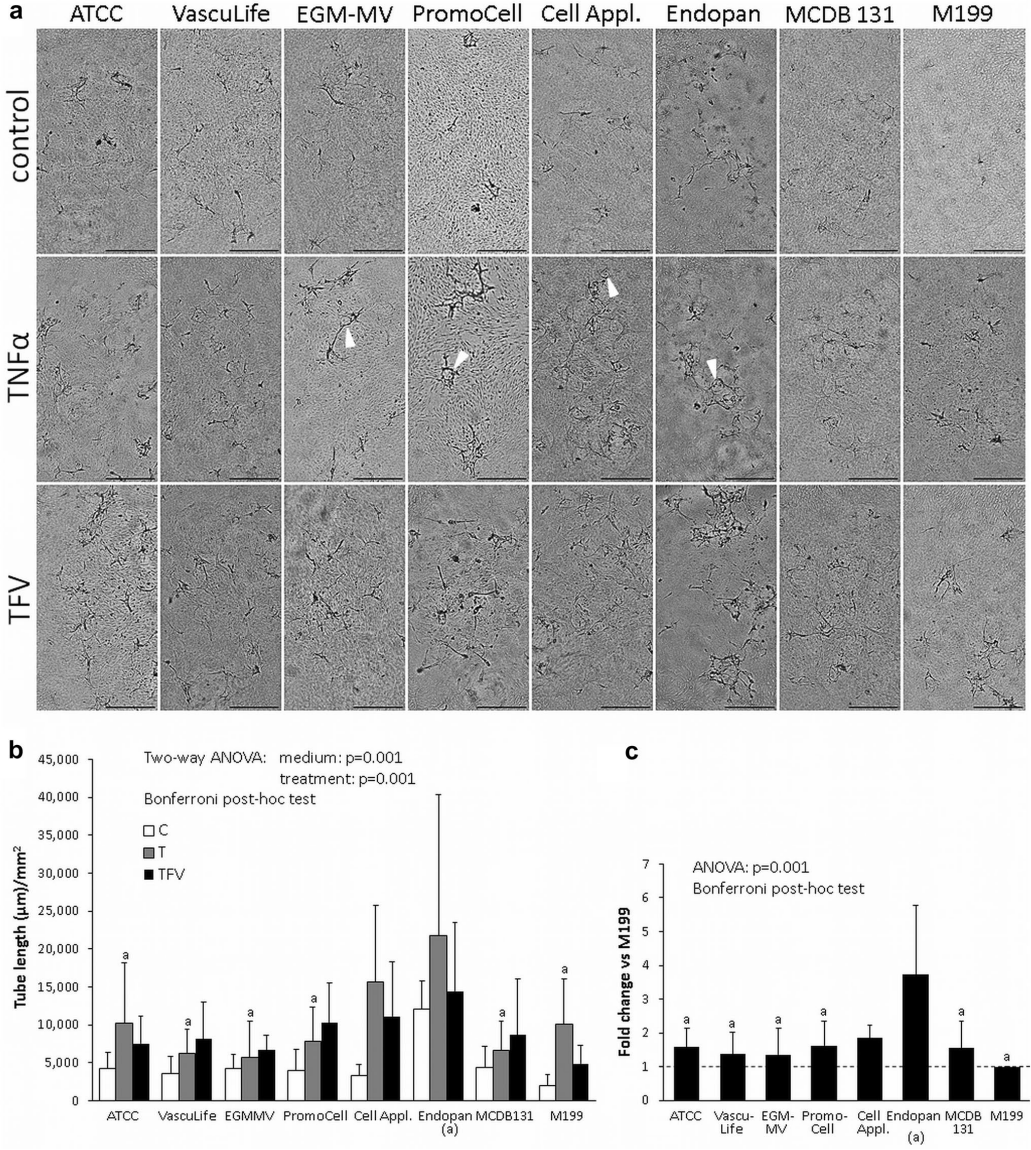
Effect of different endothelial culture media on tube formation of fpEC into a fibrin matrix. **a** Tubes formed after 24 h in the various media supplemented with 10% NBCS and 5% human serum, either without stimulation or after stimulation with T (10 ng/ml TNFα) and TFV (10 ng/ml TNFα; 10 ng/ml FGF2; 25 ng/ml VEGF). Cells were imaged using Nikon TiE-2 microscope with Andor Zyla 4.2 sCMOS camera and Nikon Plan Apoλ 4× objective. Scale bar: 500 µm. Arrowheads show the small round tubes formed by fpEC after stimulation with TNFα alone. **b** Quantification of tube formation (µm/mm^2^). **c** Normalisation of all treatments per medium to the mean tube length (µm/mm^2^) of M199 medium illustrates the similarity of data. Data are given as mean ± SD of four different fpEC isolations, each performed in triplicates. *a* significant vs respective condition in Endopan medium, *NBCS* newborn calf serum

To observe whether differences in fpEC endothelial function exist depending on the tested media, we performed fibrin in vitro angiogenesis assay. This assay enables quantification of tubes formed by endothelial cells into a 3D fibrin matrix. Routinely, we perform the assay using M199 medium, a medium free of growth factors and ECGS, supplemented with 10% NBCS and 10% human serum. This condition was used as a positive control. The various tested media were tested in their original composition (Table 2) and with additional serum supplementation (10% NBCS and 5% human serum). Both serum conditions were used unstimulated (control) as well as stimulated with TNFα (T) or with a combination of TNFα, FGF2 and VEGF (TFV). Without additional serum supplementation, no tubes were formed in any of the tested media, even when cells were stimulated with TFV (not shown). In presence of 10% NBCS and 5% human serum, cells formed tubes into the fibrin matrix. Stimulation with TNFα or with TFV increased tube formation in all media. There was no significant difference between TNFα alone, or the additional treatment with FGF2 and VEGF (Fig. 4a, b), however, morphological differences could be observed: stimulation with TNFα or TFV resulted in elongated, activated cells (Fig. 4). After treatment with TNFα, tube formation was more controlled and thus, resulted in smaller and more circular sprouts as compared to TFV. When treatments of all replicates were normalized to the mean of the tube length/mm^2^ in M199 medium, the results show that tube formation was similar in all media, except for Endopan medium, which increased tube formation by more than threefold. Interestingly, in Endopan medium, cells formed additional 2D networks on the surface of the fibrin matrix (Supplementary Fig. 2).

### Effect of culture media on fpEC phenotype

The phenotype of fpEC was investigated by assessment of presence or absence of various cell surface proteins specific for endothelial differentiation stages as well as for endothelial cell subpopulations by FACS analysis. However, phenotype of fpEC was unaffected by the culture media tested and did not differ between complex and defined media. In all media, fpEC were negative for CD45 and CD90, surface proteins expressed by hematopoietic precursors, and for the stem cell markers CD133 and CD34. However, in each medium tested, there was a small population (2.3–5.7% of total cells) positive for CD34. In all media, fpEC were positive for the endothelial cell markers CD309, CD144, CD31 and CD146 (Supplementary Fig. 3).

### Effect of culture media on ECFC colony outgrowth

Since the optimal media identified for endothelial cell proliferation and viability and the media revealing best isolatin success were different, we additionally tested the the effect of the various media on outgrowth of a further primary human endothelial cell type, i.e., endothelial colony-forming cells (ECFCs) isolated from umbilical cord blood.

**Fig. 5:**
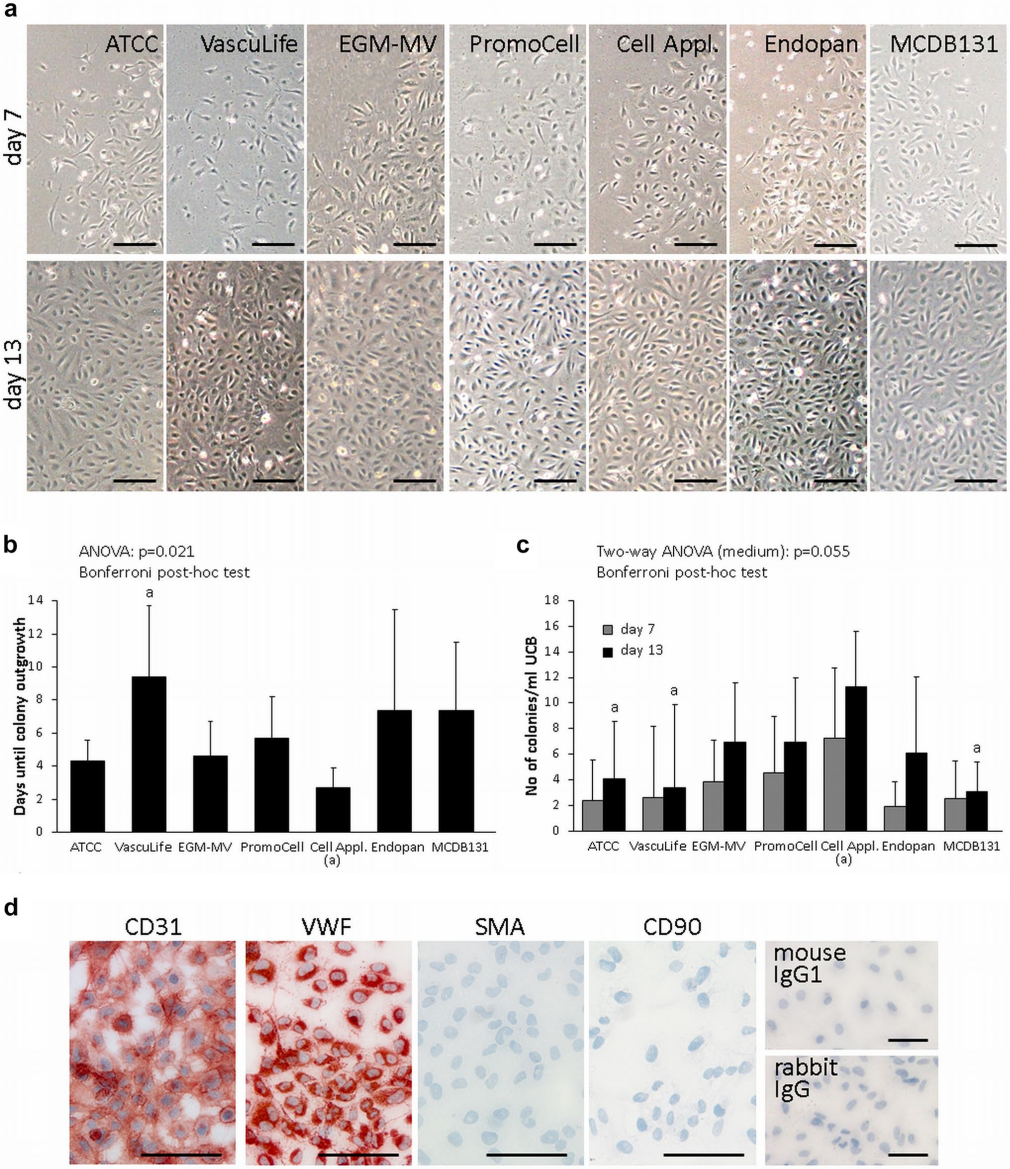
Effect of different endothelial culture media on colony outgrowth of ECFC isolated from umbilical cord blood. **a** Colony outgrowth on day 7 and day 13 after isolation. Scale bar: 200 µm. **b** Number of days until observation of first colonies after isolation. **c** Number of colonies per ml cord blood observed on day 7 and 13 after isolation. **d** Immunocytochemistry for quality control of ECFC isolations. Isolations were pure and without contaminations and thus, always positive for the endothelial cell markers CD31 and VWF and negative for the fibroblast marker CD90 and SMA. For negative controls unspecific antibodies of the same isotype were used. Scale bar: 100 µm. Data are given as mean ± SD of 5 (VascuLife; Endopan; MCDB 131) or 6 (ATCC; EGM-MV; PromoCell; Cell Applications) ECFC isolations. *a* significant vs Cell Application medium

After purification of mononucleated cells from umbilical cord blood, colony outgrowth of endothelial cells was observed in the seven different media tested above (Fig. 5a). Fastest ECFC colony outgrowth occurred in the medium from Cell Applications after 2–3 days (Fig. 5b). Also the number of colonies obtained per ml umbilical cord blood was highest in Cell Applications medium (Fig. 5c). There was no contamination outgrowth in ECFC isolations in any medium (Fig. 5d).

## Discussion

This study investigated the effect of endothelial cell culture media on outgrowth, proliferation, viability, in vitro angiogenesis and phenotype of primary human endothelial cells. Our key finding was that the optimal medium differed between colony outgrowth, proliferation and in vitro angiogenesis, highlighting the distinct demands of endothelial cells depending on the different processes and functions.

Specifically isolation success and endothelial cell outgrowth are decisive for subsequent endothelial cell culture. In fact, colony outgrowth after isolation differed between the media: we did not succeed in isolation of persistent fpEC colonies in MCDB 131 medium, whilst in medium from PromoCell, ATCC and Cell Applications, frequently propagating fpEC colonies grew out. Least contaminations grew out in PromoCell medium. Besides the promotion of endothelial cell growth, growth factors and growth supplements present in the media may as well support outgrowth of contaminations. Certain agents, however, can be used to specifically block outgrowth of some cell types. For instance, the replacement of l-valine by d-valine, or the addition of Cis-OH-proline hinders fibroblast outgrowth (Gilbert and Migeon 1975; Kao and Prockop 1977). The presence of such fibroblast growth inhibitors though was not implicated in any of the tested media.

Also morphology of new fpEC colonies was susceptible to medium composition as reflected by a distinct, more network-like growing pattern at the colony borders in VascuLife medium. After reaching confluency, however, this growing pattern disappeared and growth of cells in all media was similar. Surprisingly, the medium from ATCC, with a composition very similar to VascuLife medium, did not induce such colony morphology, indicating that already subtle differences in medium composition affect endothelial growth.

The isolation of primary endothelial cells is a susceptible procedure and isolation success depends on various biological and technical factors. Our isolation protocol for fpEC is standardized with consistent perfusion pressure and perfusion time of the vessels, but various parameters cannot be controlled for: the interaction between endothelial cells and ECM may vary in between individuals and in between vessels of the same individual, thus increasing the variance in colony number. Due to the variance of the data, the analysis determining the effect of culture media on isolation success of fpEC was underpowered. Sample size calculation revealed that 20 independent cell isolations would be required to identify a statistical significant difference in fpEC colony outgrowth between the media from VascuLife and Cell Applications, i.e., the medium with the lowest colony outgrowth vs the medium with the highest number of colonies. Thus, lack of statistical significance does not prove that isolation is unaffected by the media used.

Thus, in order to verify the media effect on endothelial cell outgrowth in a different primary endothelial cell phenotype, we additionally tested the media on outgrowth of umbilical cord blood-derived ECFCs. Whilst mature fpEC were enzymatically detached from the chorionic vessels and isolations were sometimes contaminated with other cell types that are also present in the vessel wall, umbilical cord blood-derived ECFC isolations were free of any contaminations and constantly gave rise to propagating endothelial cell isolations. Fastest colony outgrowth and most colonies occurred in Cell Applications medium. This finding parallels data from fpEC which also, although without reaching significance, revealed fastest outgrowth in Cell Applications medium. When outgrowth of both cell types, i.e., fpEC and ECFCs, was analysed together, combined analysis highlighted the common effect (Supplementary Fig. 4). The medium from Cell Applications is a defined medium containing a combination of EGF, FGF2 and VEGF. Specifically, VEGF promotes outgrowth of endothelial cell colonies from circulating endothelial progenitor cells (d’Audigier et al. 2014) and thus, may be important for the attachment of single cells and the initiation of proliferation.

In contrast to colony outgrowth, proliferation of established fpEC isolations was particularly supported by complex media containing ECGS, and best results were obtained in media from VascuLife and ATCC. Both are supplemented with ECGS, ascorbic acid and heparin. This parallels results from Proulx et al. (2007) and Terramani et al. (2000) who tested different media supplements such as EGF, ascorbic acid, chondroitin sulphate and ECGS on proliferation of porcine corneal endothelial cells (Proulx et al. 2007), and ECM (extracellular matrix) coating, mitogens, heparin and ECGS on HUVEC (Terramani et al. 2000), respectively. Both studies also identified a positive effect of ECGS, ascorbic acid and heparin on cell proliferation, suggesting that the favourable effect of complex media on proliferation may be a general finding in endothelial cells and not restricted to fpEC.

Angiogenesis is a multistep process including matrix degradation and migration of cells. In vitro angiogenesis assay revealed that most tubes were formed in Endopan medium, a defined medium supplemented with the highest number of individual growth factors, i.e., EGF, FGF2, VEGF and IGF1, although with unknown concentration. The fact that in Endopan medium cells formed an additional 2D network on top of the fibrin matrix further highlights the particular pro-angiogenic composition of this medium. The addition of TNFα increased tube formation in all media, but not in all media the pro-angiogenic effect was additionally increased with FGF2 and VEGF. This may be due to the fact that some media were already supplemented with FGF2 and VEGF. For experimental application of fibrin angiogenesis, however, media which do not over-stimulate tube formation and thus, retain the cells’ sensitivity towards the action of additional factors are preferred.

The complex composition of media comprising growth factors, hormones and other regulatory molecules may indicate a modulatory effect of these media not only on outgrowth, proliferation and in vitro angiogenesis, but also on the phenotype of the cells. However, phenotype of fpEC was unaffected by media composition.

Overall, our data demonstrate that isolation success and behaviour of primary endothelial cells depend on the culture medium and on the composition and nature of supplements. The susceptibility of endothelial cells to soluble factors is also indicated by various studies revealing modulated growth and function of endothelial cells when culture medium was conditioned by other cell types and thus, enriched with certain secreted molecules (Di Santo et al. 2014; Gharaei et al. 2018; Merfeld-Clauss et al. 2015). Our study further highlights that colony outgrowth of freshly isolated cells vs proliferation of established endothelial cell cultures may differ depending on demands on the culture medium.

## Electronic supplementary material

Below is the link to the electronic supplementary material.

Supplementary material 1 (PPTX 2481 kb)

Supplementary material 2 (PPTX 1037 kb)

Supplementary material 3 (PPTX 1320 kb)

Supplementary material 4 (PPTX 36 kb)

## References

[CR1] Aitsebaomo J, Portbury AL, Schisler JC, Patterson C (2008). Brothers and sisters: molecular insights into arterial-venous heterogeneity. Circ Res.

[CR2] Carmeliet P (2005). Angiogenesis in life, disease and medicine. Nature.

[CR3] d’Audigier C, Gautier B, Yon A (2014). Targeting VEGFR1 on endothelial progenitors modulates their differentiation potential. Angiogenesis.

[CR4] Di Santo S, Seiler S, Fuchs AL, Staudigl J, Widmer HR (2014). The secretome of endothelial progenitor cells promotes brain endothelial cell activity through PI3-kinase and MAP-kinase. PLoS One.

[CR5] Engelmann K, Friedl P (1989). Optimization of culture conditions for human corneal endothelial cells. Vitr Cell Dev Biol.

[CR6] Friedl P, Tatje D, Czpla R (1989). An optimized culture medium for human vascular endothelial cells from umbilical cord veins. Cytotechnology.

[CR7] Ganguly A, Zhang H, Sharma R, Parsons S, Patel KD (2012). Isolation of human umbilical vein endothelial cells and their use in the study of neutrophil transmigration under flow conditions. J Vis Exp.

[CR8] Gharaei MA, Xue Y, Mustafa K, Lie SA, Fristad I (2018). Human dental pulp stromal cell conditioned medium alters endothelial cell behavior. Stem Cell Res Ther.

[CR9] Gilbert SF, Migeon BR (1975). d-valine as a selective agent for normal human and rodent epithelial cells in culture. Cell.

[CR10] Kao WW, Prockop DJ (1977). Proline analogue removes fibroblasts from cultured mixed cell populations. Nature.

[CR11] Koolwijk P, van Erck MG, de Vree WJ (1996). Cooperative effect of TNFalpha, bFGF, and VEGF on the formation of tubular structures of human microvascular endothelial cells in a fibrin matrix. Role of urokinase activity. J Cell Biol.

[CR12] Lang I, Schweizer A, Hiden U (2008). Human fetal placental endothelial cells have a mature arterial and a juvenile venous phenotype with adipogenic and osteogenic differentiation potential. Differentiation.

[CR13] Loegl J, Nussbaumer E, Hiden U (2016). Pigment epithelium-derived factor (PEDF): a novel trophoblast-derived factor limiting feto-placental angiogenesis in late pregnancy. Angiogenesis.

[CR14] Loegl J, Nussbaumer E, Cvitic S, Huppertz B, Desoye G, Hiden U (2017). GDM alters paracrine regulation of feto-placental angiogenesis via the trophoblast. Lab Investig.

[CR15] Merfeld-Clauss S, Lupov IP, Lu H, March KL, Traktuev DO (2015). Adipose stromal cell contact with endothelial cells results in loss of complementary vasculogenic activity mediated by induction of activin A. Stem Cells.

[CR16] Minehata K, Mukouyama YS, Sekiguchi T, Hara T, Miyajima A (2002). Macrophage colony stimulating factor modulates the development of hematopoiesis by stimulating the differentiation of endothelial cells in the AGM region. Blood.

[CR17] Naschberger E, Regensburger D, Tenkerian C (2018). Isolation of human endothelial cells from normal colon and colorectal carcinoma—An improved protocol. J Vis Exp.

[CR18] Peh GS, Toh KP, Ang HP, Seah XY, George BL, Mehta JS (2013). Optimization of human corneal endothelial cell culture: density dependency of successful cultures in vitro. BMC Res Notes.

[CR19] Proulx S, Bourget JM, Gagnon N (2007). Optimization of culture conditions for porcine corneal endothelial cells. Mol Vis.

[CR20] Rakocevic J, Orlic D, Mitrovic-Ajtic O (2017). Endothelial cell markers from clinician’s perspective. Exp Mol Pathol.

[CR21] Tasev D, Dekker-Vroling L, van Wijhe M, Broxterman HJ, Koolwijk P, van Hinsbergh VWM (2018). Hypoxia impairs initial outgrowth of endothelial colony forming cells and reduces their proliferative and sprouting potential. Front Med (Lausanne).

[CR22] Terramani TT, Eton D, Bui PA, Wang Y, Weaver FA, Yu H (2000). Human macrovascular endothelial cells: optimization of culture conditions. Vitr Cell Dev Biol Anim.

[CR23] Timmermans F, Plum J, Yoder MC, Ingram DA, Vandekerckhove B, Case J (2009). Endothelial progenitor cells: identity defined?. J Cell Mol Med.

[CR24] Wang J, Bian Y, Wang Z (2014). MicroRNA-152 regulates DNA methyltransferase 1 and is involved in the development and lactation of mammary glands in dairy cows. PLoS One.

[CR25] Zabini D, Nagaraj C, Stacher E (2012). Angiostatic factors in the pulmonary endarterectomy material from chronic thromboembolic pulmonary hypertension patients cause endothelial dysfunction. PLoS One.

