# Supplementary material for: Outgrowth, proliferation, viability, angiogenesis and phenotype of primary human endothelial cells in different purchasable endothelial culture media: feed wisely
Source: Histochem Cell Biol. 2019 Sep 21;152(5):377–90. doi: 10.1007/s00418-019-01815-2 (PMC6842357; doi:10.1007/s00418-019-01815-2)
Supplement: Supplementary file 1 — Supplementary material 1 (PPTX 2481 kb) [file 418_2019_1815_MOESM1_ESM.pptx]

## Slide 1
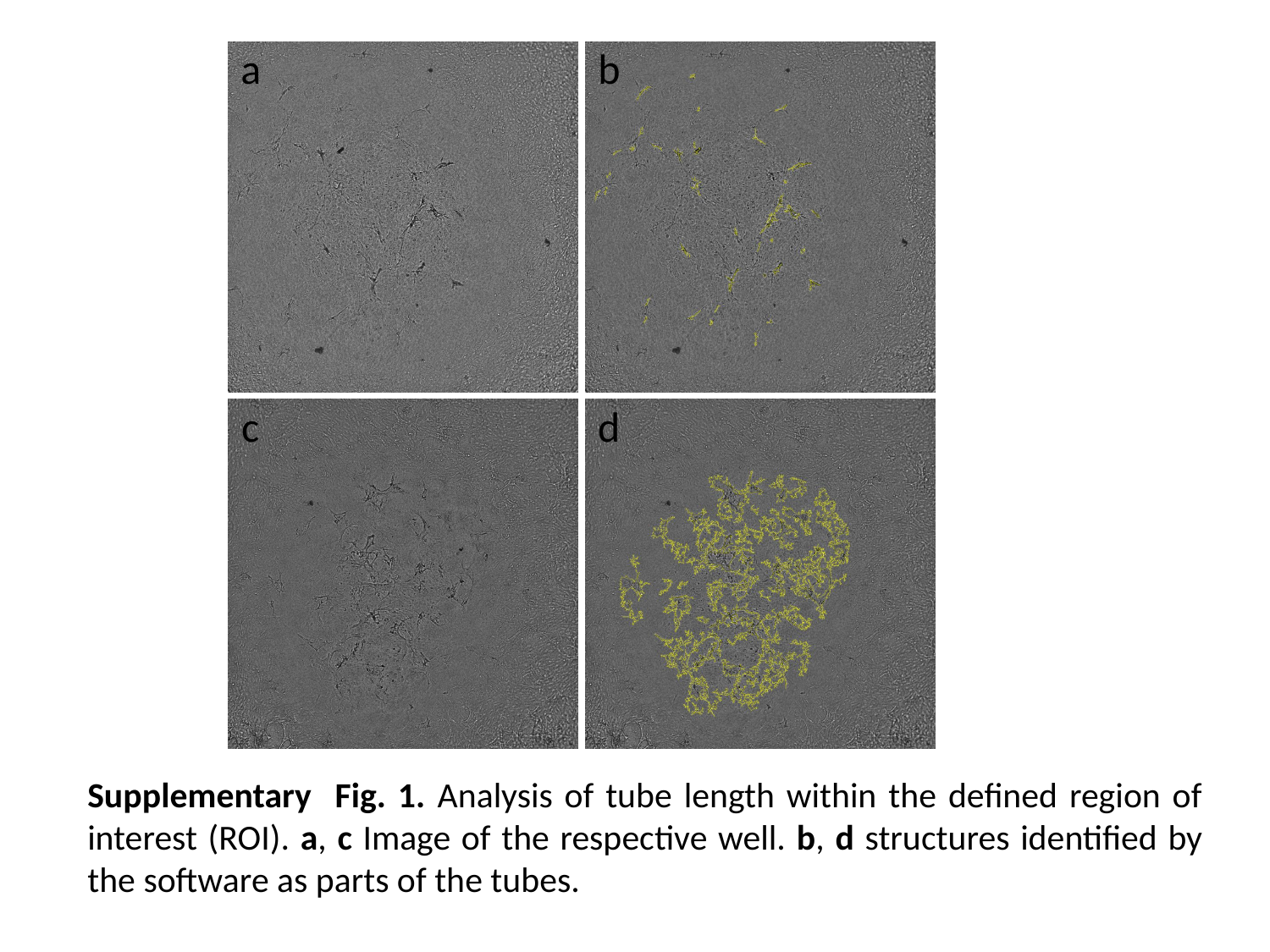

a
b
c
d
Supplementary Fig. 1. Analysis of tube length within the defined region of interest (ROI). a, c Image of the respective well. b, d structures identified by the software as parts of the tubes.
