# Supplementary material for: Outgrowth, proliferation, viability, angiogenesis and phenotype of primary human endothelial cells in different purchasable endothelial culture media: feed wisely
Source: Histochem Cell Biol. 2019 Sep 21;152(5):377–90. doi: 10.1007/s00418-019-01815-2 (PMC6842357; doi:10.1007/s00418-019-01815-2)
Supplement: Supplementary file 2 — Supplementary material 2 (PPTX 1037 kb) [file 418_2019_1815_MOESM2_ESM.pptx]

## Slide 1
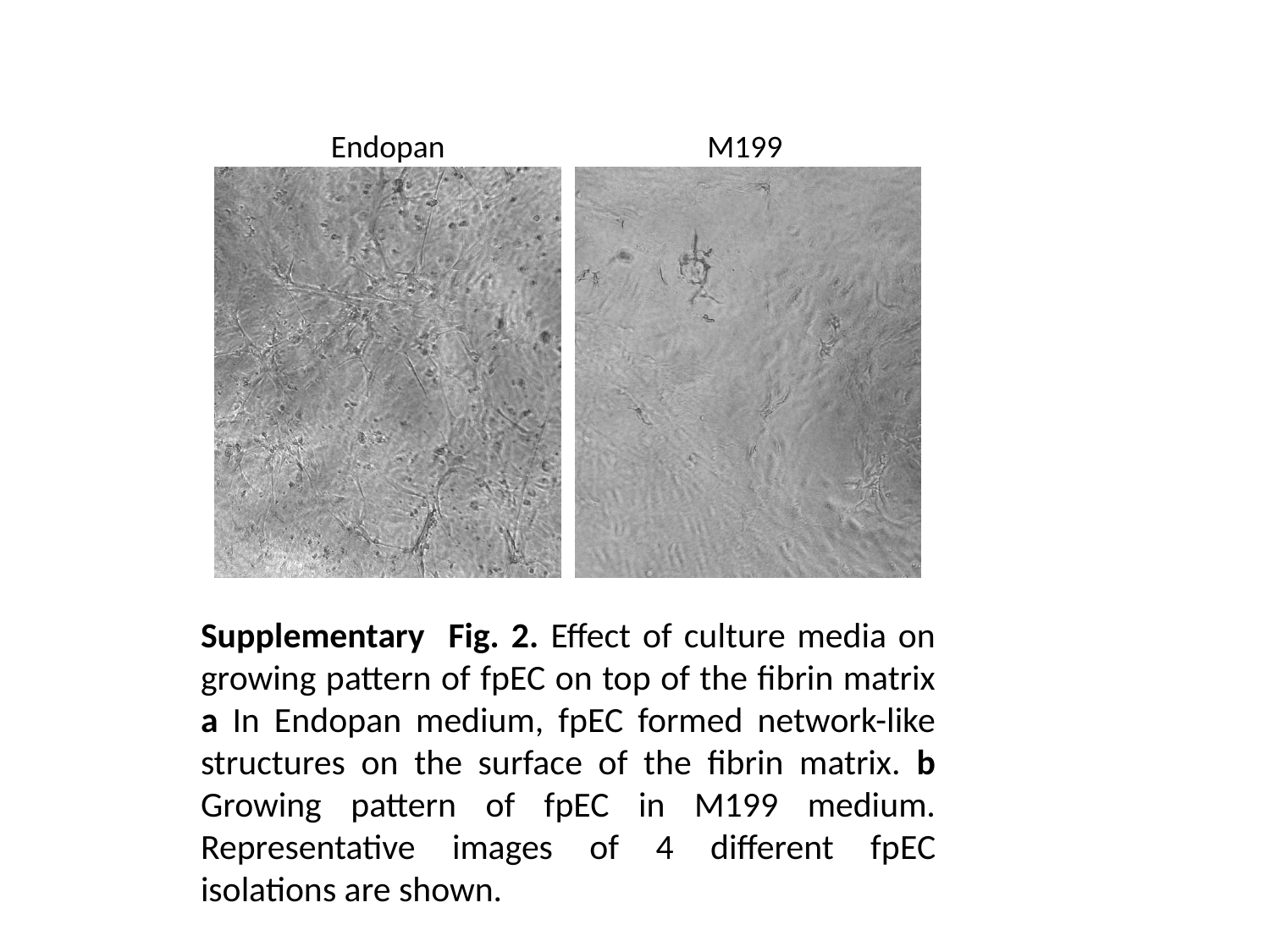

Endopan
M199
Supplementary Fig. 2. Effect of culture media on growing pattern of fpEC on top of the fibrin matrix a In Endopan medium, fpEC formed network-like structures on the surface of the fibrin matrix. b Growing pattern of fpEC in M199 medium. Representative images of 4 different fpEC isolations are shown.
