# Supplementary material for: Outgrowth, proliferation, viability, angiogenesis and phenotype of primary human endothelial cells in different purchasable endothelial culture media: feed wisely
Source: Histochem Cell Biol. 2019 Sep 21;152(5):377–90. doi: 10.1007/s00418-019-01815-2 (PMC6842357; doi:10.1007/s00418-019-01815-2)
Supplement: Supplementary file 3 — Supplementary material 3 (PPTX 1320 kb) [file 418_2019_1815_MOESM3_ESM.pptx]

## Slide 1
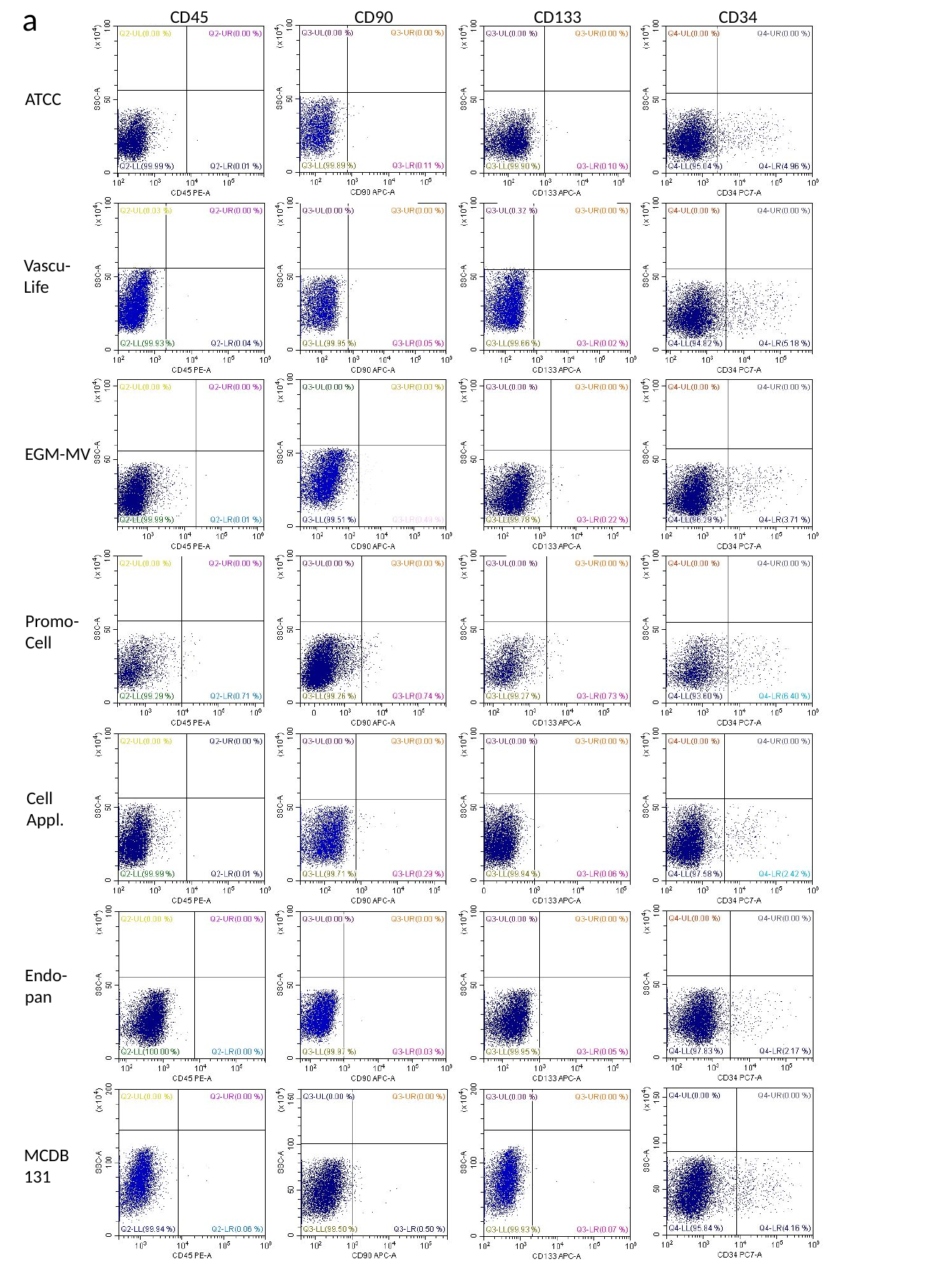

a
CD133
CD90
CD34
CD45
ATCC
Vascu-
Life
EGM-MV
Promo-
Cell
Cell
Appl.
Endo-
pan
MCDB
131

## Slide 2
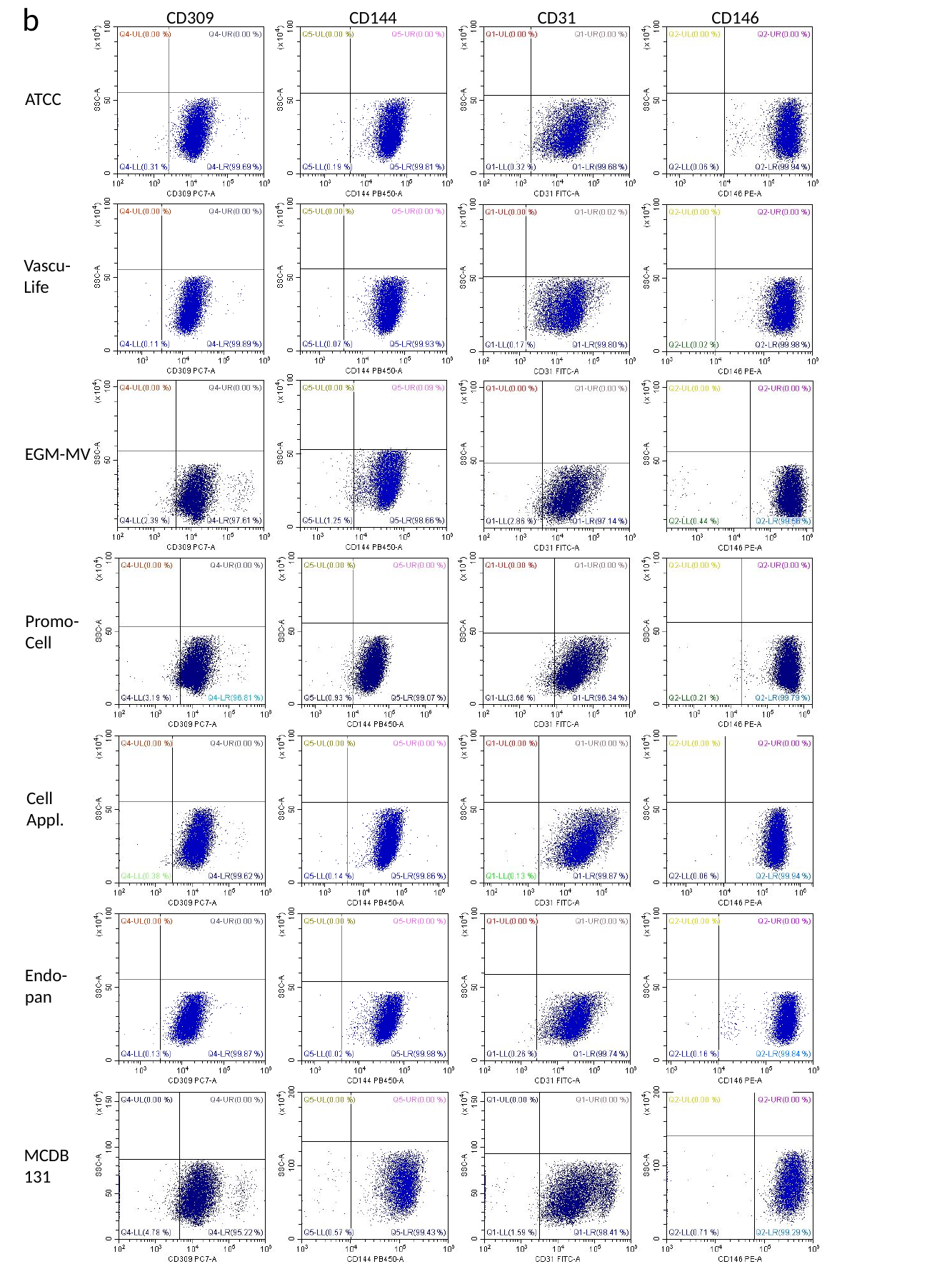

b
CD144
CD146
CD309
CD31
ATCC
Vascu-
Life
EGM-MV
Promo-
Cell
Cell
Appl.
Endo-
pan
MCDB
131

## Slide 3
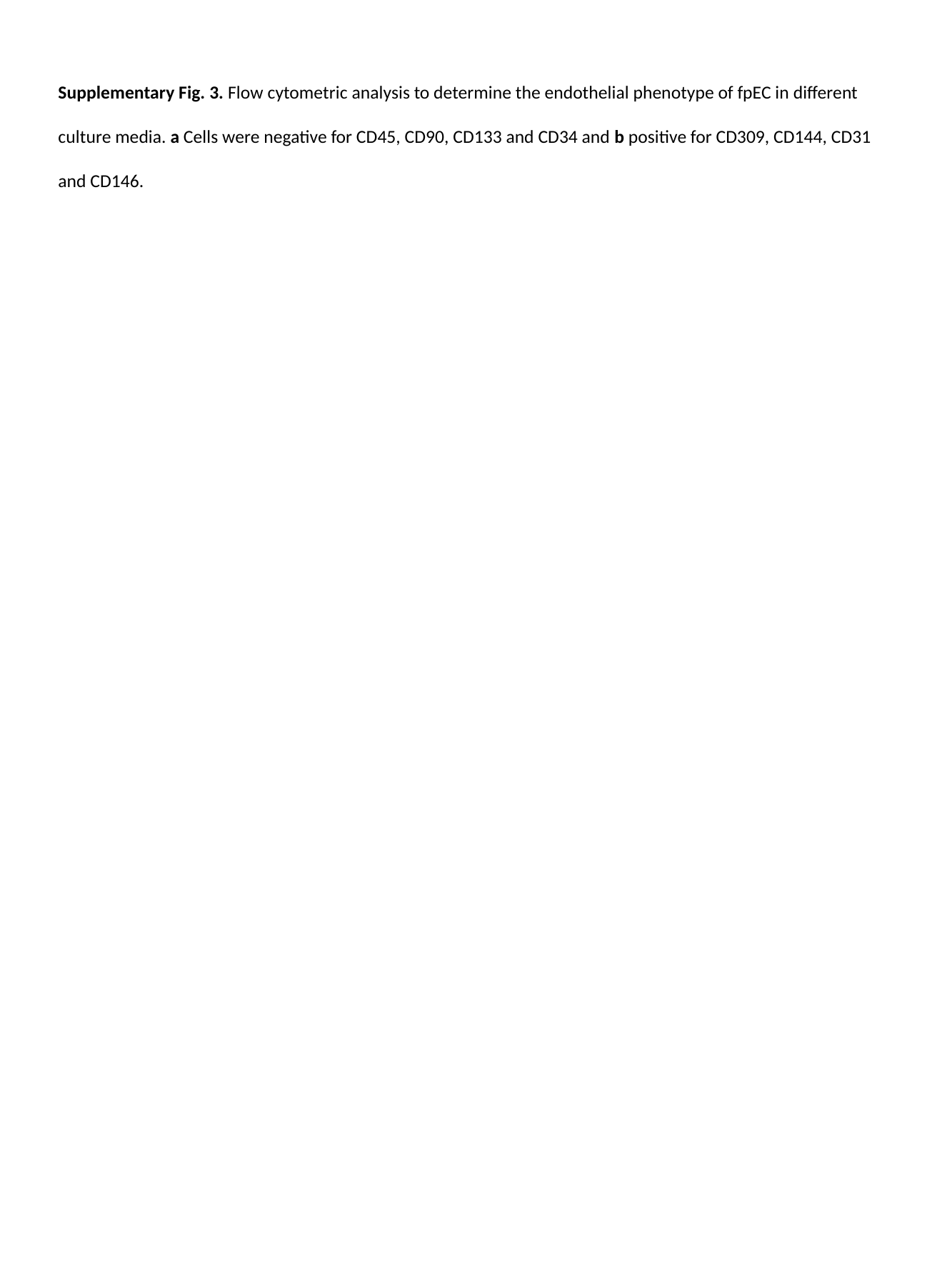

# Supplementary Fig. 3. Flow cytometric analysis to determine the endothelial phenotype of fpEC in different culture media. a Cells were negative for CD45, CD90, CD133 and CD34 and b positive for CD309, CD144, CD31 and CD146.
