# Supplementary material for: Outgrowth, proliferation, viability, angiogenesis and phenotype of primary human endothelial cells in different purchasable endothelial culture media: feed wisely
Source: Histochem Cell Biol. 2019 Sep 21;152(5):377–90. doi: 10.1007/s00418-019-01815-2 (PMC6842357; doi:10.1007/s00418-019-01815-2)
Supplement: Supplementary file 4 — Supplementary material 4 (PPTX 36 kb) [file 418_2019_1815_MOESM4_ESM.pptx]

## Slide 1
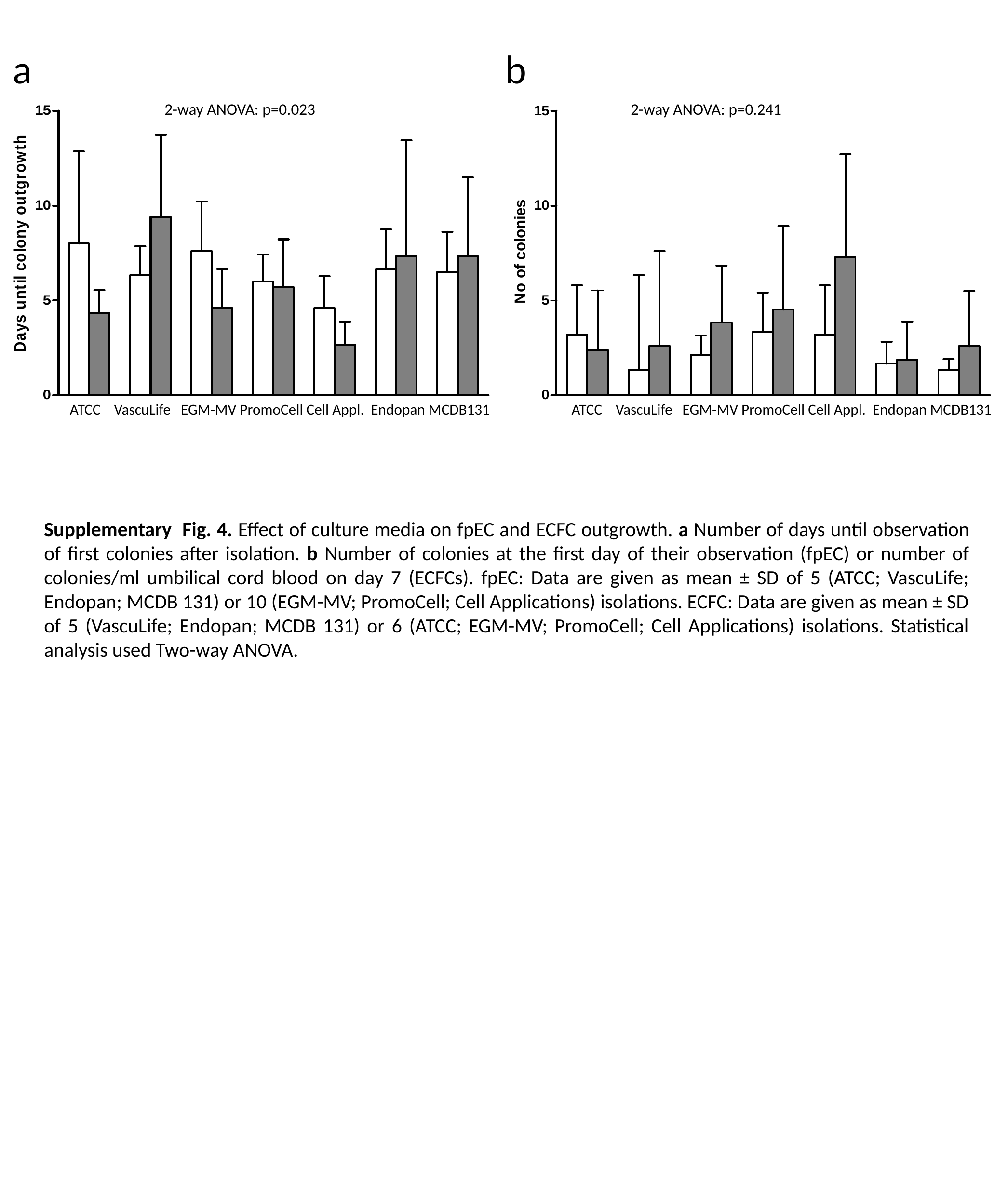

b
a
2-way ANOVA: p=0.023
2-way ANOVA: p=0.241
 ATCC VascuLife EGM-MV PromoCell Cell Appl. Endopan MCDB131
 ATCC VascuLife EGM-MV PromoCell Cell Appl. Endopan MCDB131
Supplementary Fig. 4. Effect of culture media on fpEC and ECFC outgrowth. a Number of days until observation of first colonies after isolation. b Number of colonies at the first day of their observation (fpEC) or number of colonies/ml umbilical cord blood on day 7 (ECFCs). fpEC: Data are given as mean ± SD of 5 (ATCC; VascuLife; Endopan; MCDB 131) or 10 (EGM-MV; PromoCell; Cell Applications) isolations. ECFC: Data are given as mean ± SD of 5 (VascuLife; Endopan; MCDB 131) or 6 (ATCC; EGM-MV; PromoCell; Cell Applications) isolations. Statistical analysis used Two-way ANOVA.
